# Construction of Self-defensive Antibacterial and Osteogenic AgNPs/Gentamicin Coatings with Chitosan as Nanovalves for Controlled release

**DOI:** 10.1038/s41598-018-31843-2

**Published:** 2018-09-07

**Authors:** Wenhao Zhou, Yangyang Li, Jianglong Yan, Pan Xiong, Qiyao Li, Yan Cheng, Yufeng Zheng

**Affiliations:** 1 Academy for Advanced Interdisciplinary Studies, Peking University, Beijing, 100871 China; 2 Department of Advanced Materials and Nanotechnology, College of Engineering, Peking University, Beijing, 100871 China; 3 Department of Biomedical Engineering Materials Research Institute, The Huck Institutes of the Life Sciences, The Pennsylvania State University, University Park, State College, PA 16802 USA

**Keywords:** Biomaterials, Cell adhesion

## Abstract

To solve the Ti implants-associated infection and poor osseointegration problems, we have constructed the AgNPs/gentamicin (Gen)-loaded silk fibroin (SF) coating with acceptable antibacterial and osteogenic aptitude. Nevertheless, due to uncontrollably sustained drug release, this bactericidal coating encountered some tricky problems, such as local high Ag concentration, short life-span and potential cytotoxicity. In this work, a chitosan (CS) barrier layer was constructed to prebuilt the SF-based film by two means, dip-coating (DCS) and spin-coating (SCS). Intriguingly, the CS barrier layer constructed by spin-coating highly improved the hydrophilic and protein-absorbed performances. As verified in the release profile, both coatings showed a prolonged and pH-dependent pattern of Ag^+^ with an accelerated release in acidic condition. Also, the multilayer coating with a SCS barrier layer showed an apparent bacteria-trigged antibacterial and biofilm-inhibited performances, whereas the improvements of antibacterial abilities of DCS coating were limited. The mechanisms could be explained that the pH decrease induced by the attachment and proliferation of bacteria triggered collapse of CS barrier layer, accelerating the release of bactericides. Moreover, benefitted from pH-dependent release behavior of Ag and bioactive SCS layer, functional coatings highly enhanced the initial adhesion, migration and proliferation of preosteoblast MC3T3-E1 cells, and subsequently accelerated osteoblast differentiation (alkaline phosphatase production). A relevant aspect of this work was to demonstrate the essential effect of reasonable construction of self-defensive barrier layer in achieving the balance between the high-efficiency bacterial killing and osteogenic activity, and highlighted its excellent potential in clinical applications.

## Introduction

Biomaterial-associated infections (BAI) is a widespread and rapidly growing problem^1–4^, whereas the traditional treatment with systemic antibiotics is often inefficient due to the formation of bacterial biofilms, in which bacteria are poorly responsive to bactericides^5–8^. Therefore, the construction of antibacterial surface to prevent bacterial colonization at early stages is regarded as a crucial pathway to solve BAI problems. To inhibit the initial bacterial adhesion, functional surfaces of biomedical devices are well designed by variations of surface nano- and microtopography^9,10^, creating antifouling coatings *via* surface modification with hydrophilic polymers^11^, or developing cationic coatings which kill bacteria on contact^12,13^.

In prior work, we have built Ag nanoparticles (AgNPs)/gentamicin (Gen)-loaded silk fibroin (SF)-based biomimetic coatings on orthopedic titanium, showing acceptable antibacterial and osteogenetic abilities^14^. Even so, the burst initial release of bactericides and long-term low-concentration release, which easily induced the emergence of antibiotics-resistance bacteria and caused potential cytotoxicity, strictly restricted its biomedical applications. Therefore, the targeted and controlled release of therapeutic agents from intelligent coatings, which can be realized by responding to various environmental stimuli like pH, is a promising approach to mitigate the toxicity issue and retard premature depletion of the drug supply/reservoir^15–17^.

Different from synthetic polyelectrolytes, such as poly (carboxylic acid)s^18^, used earlier for constructing pH-responsive coatings, as a natural and biocompatible polymer, CS provides a favorable pH-responsive property^7,19–21^. CS is a natural cationic polysaccharide that is composed of *β*-(1–4)-link ked glucosamine units, together with some *N-*acetyl-D-glucosamine units, and it is commercially obtained by the exhaustive deacetylation of chitin^22^. Specifically, the amino groups of CS can be protonated and deprotonated with the change of pH, revealing a possible pathway for utilizing CS to construct intelligent coatings that respond to an external pH-stimuli. Moreover, with a pKa value around 6.5, CS is insoluble in neutral and alkaline solutions, but it is soluble in acidic medium, allowing it to be processed under mild conditions. The CS layer has been broadly applied to build pH-responsive nanovalves to control the release of the drug^23–26^. With regard to biocompatible and osteoinductive considerations, CS coating composed with large amounts of cationic groups can electrostatically connect with anionic glycosaminoglycans (GAGs) which contains a large number of cytokines/growth factors. A coating containing CS may therefore retain and concentrate growth factors secreted by the colonizing cells^27^. CS coating has been found to promote cell growth and mineral-rich matrix deposition by co-culturing with osteoblasts and showed positive influences on osteogenesis *in vitro* and *in vivo*^28^. Human osteoblasts on the surface of CS layer maintained a spherical morphology and showed high type I collagen expression^29^. CS-contained coating also promoted the differentiation of osteoprogenitor cells and bone formation^30–32^. In recent years, researchers focused more on the applications of CS and SF mixtures in biofunctional scaffolds and coatings, which showed highly acceptable biocompatibility^33,34^.

Here, a CS barrier layer was constructed by two means (dip-coating and spin-coating) to cover AgNPs/Gen-loaded SF-based inner layer, forming a pH-dependent multilayer coating with pH-triggered antibacterial and osteoinductive properties. The aims of the present work were: (1) to develop and characterize the functional multilayer coating; (2) to assess the pH-dependent release behavior of Ag^+^; (3) to verify that local acidification caused by secretion of multiplied bacteria could lead to the CS barrier layer collapse and accelerate release of bactericides, eradicating anchored bacteria; (4) to evaluate the *in vitro* attachment, spread and proliferation of osteoblasts (MC3T3-E1) on the surface of multilayer coatings, and ALP expression, mineral deposition and collagen secretion were also examined to reveal the osteoinductive ability.

## Results and Discussion

### Fabrication and characterization of CS-decorated coating

The surface morphologies and microstructures of the multilayer coating were obtained by SEM observation. Compared to the rough topography of the pure Ti surface, in which the abundant parallel scratches are caused by mechanical polish (Fig. 2a1), PD layer displayed a relatively flat surface with less flaws (Fig. 2a2). Intriguingly, after deposition of DLSF layer, the surface became more smooth and compact, and a mass of Ag nanoparticles uniformly distributed on the surface (Fig. 2a3).

As showed in TEM images (Fig. S1), the diameter of AgNPs was around 10 nm. Almost no particles were observed on Ti-PD-DLSF-DCS and Ti-PD-DLSF-SCS due to the further coverage of CS nanovalves layer. Moreover, Ti-PD-DLSF-SCS displayed a more even surface with some small cracks.

An overall understanding of the surface chemical property of biomaterials, such as functional groups and chemical components, is crucial to disclose the responsive behaviors of osteoblastic cells and bacteria, which ultimately affects the success of implanting operation. In detail, the surface chemical properties of CS-decorated coatings were investigated by FTIR and XPS. No FTIR characteristic peak of PD was observed due to the thickness was too thin, but the disappearance of some Ti characteristic peaks in the line of Ti-PD was an evidence of PD layer (Fig. 2b, blue circle). In the FTIR spectra of DLSF coating, the peaks at 1655 cm^−1^, 1540 cm^−1^, and 1250 cm^−1^ corresponded to the vibrational transition bands of C=O stretching (amide I), N-H de-formation and C–N stretching (amide II), and C–N stretching and N–H deformation (amide III), implying the coexistence of α-helix and β-sheet structures. As confirmed, Tyr residuals of DLSF could reduce Ag^+^ ions into Ag nanoparticles by assistance of UV irradiation. The relatively weak peaks at 3434 cm^−1^, 1610 cm^−1^ and 1114 cm^−1^ were ascribed to the existence of AgNPs in DLSF layer. The broad peak at 3434 cm^−1^ was referred as the strong stretching vibrations of O-H, representing the bonding between AgNPs and SF. Moreover, the band at 1610 cm^−1^ was identified as C-C stretching vibrations and the characteristic peak at 1114 cm^−1^ was ascribed to amide-II, which were responsible for the reduction of metal ions by SF. Due to the low amount, there was no characteristic peak for Gen. The main representative peaks in spectrum of Ti-PD-DLSF-DCS and Ti-PD-DLSF-SCS were similar, and the absorption peaks at 1584 cm^−1^, 1075 cm^−1^ and 1035 cm^−1^, related to amine II, C–O stretching and O–H bending, respectively.

Furtherly, the photoelectron spectra of the wide-scan XPS could evidence the specific chemical components of multilayer coatings. As shown in Fig. 2c, besides C, O, and N elements, two distinct doublet peaks at round 370 eV were observed in Ti-PD-DLSF, representing the existence of Ag(0) 3d_3/2_ and Ag(0) 3d_5/2_. Notably, the good chemical stability of AgNPs was probably attributed to their high crystallinity and protection of SF, a certified stabilizer^35^, which was very important for their antibacterial activities. Furthermore, about 0.28% Ag existed in Ti-PD-DLSF, while only 0.04% and 0.02% Ag was detected after deposition and spinning of CS barrier layer. As shown in Fig. 2e, higher proportion of C-N and C=O bonds was observed in Ti-PD-DLSF-SCS group, representing more active terminal groups and more close combination with DLSF layer.

Additionally, the contact angle (CA) measurements were utilized to assess the wettability of the multilayer coatings. As shown in Fig. 3a, the bare Ti was less hydrophilic (49.6°) compared to PD layer(14.2°). In accordance with our previous results^14^, DLSF layer exhibited the CA value of 40.9°, largely attributed to the content of β sheet structure. The SCS barrier layer displayed the best hydrophilicity (28.5°), owing to the presence of abundant hydrophilic protonated amino groups and the flat surface.

After biomedical devices are implanted into body, the first event taking place at the biomaterial-tissue interface is protein adsorption, playing a critical role in the subsequent cell behaviors like adhesion, proliferation and differentiation. Therefore, a nonspecific FITC-labeled BSA was employed to evaluate the bioaffinity of multilayer coatings *in vitro*. Fig. 3b and c displayed the quantitative and qualitative results of adhesive albumin in the initial 60 min. As a consequence of low-charge surface, both pure Ti and PD layer exhibited relatively weak and uniform green fluorescence on the surface. With regard to DLSF layer, some aggregates with high fluorescence intensity emerged in the surface, due to the good bioaffinity of SF, a kind of natural proteins. Notably, both PD-DLSF-DCS and PD-DLSF-SCS coatings displayed better protein-adsorbed properties, according to the quantitative calculation (p < 0.05), ascribed to the excellent bioaffinity of CS layer, which tended to combine with various ECM proteins such as fibronectin, glycoprotein and growth factors. One possible explanation was that abundant amino groups in CS layer provided plentiful active binding sites for bioactive molecules with thiols or primary amines moieties. Taken together, the data of FTIR, XPS, CA and protein adsorption indicated the successful interfacial assembly of multilayer coatings by two means (dip-coating and spin-coating), and spin-coating showed the best bioactive properties.

### pH-responsive drug release

The ideal drug delivery coatings required “low-rate release” before local acidification, induced by bacteria invasion. Thus the samples were immersed in buffer solutions with different pH values to evaluate their release profiles (Fig. 4). As shown in non-cumulative release curve, SCS-decorated coatings showed an initial burst release of Ag^+^ in all pH conditions due to the extensive concentration gradient, and then sustained slow release over an extended period of time. However, the initial release amount occupied a small fraction of total laden Ag^+^. As shown in Fig. 4b and c, the release of Ag^+^ from the multilayer coatings was pH-dependent, and the release rate increased with the decrease of pH value. In physiological condition (pH 7.5), the amount of the released Ag^+^ from PD-DLSF-SCS was lower than 30% after 14 d immersion. Oppositely, when the medium turned to be acidic, the release rate obviously increased. Throughout the entire period, approximately 54.6% and 74.2% of the entrapped Ag was released in medium at pH 5.5 and pH 3.5, due to the protonated in acidic condition caused collapse of CS barrier layer and the electrostatic repulsion with Ag^+^. However, the DCS-decorated multilayer coating showed very similar release curve, and slightly worse pH-responsive property compare with SCS group, and related data were put in Fig. S2. To some extent, external pH changes also had influences on inner SF layer property. As proved, SF-based coatings and nanoparticles could serve as pH-dependent drug delivery platform, due to charge and conformation changes^36^. In our study, the pH-dependent capability of SF layer was also investigated (Fig. S2). But it was worth noting that even at neutral medium, approximate 48% Ag^+^ released from SF coating without CS barrier layer, which would cause high cytotoxicity and induce bacterial resistance. So, the outmost CS barrier layer was well-designed and essential to realize pH-dependent release and inhibit the initial burst release of Ag^+^.

### pH-dependent antibacterial property of multilayer coating

Actually, ideal drug-loaded coatings presented “release-inhibition” pattern at physiological pH and became “open-state” to release drug only when and where bacteria multiplied on the surface, inducing decrease of local pH value. Based on this concept, a CS barrier layer as nanovalve was introduced to decorate AgNPs/Gen-loaded SF coating, in which dense and hydrophobic CS layer blocked the release of bactericides at normal physiological conditions. When bacteria colonized on the surface, the local acidification induced by bacterial metabolism allowed the CS barrier layer to collapse due to mutual repulsion between protonated amino groups of CS chains, and further accelerated the release of AgNPs/Gen complexes.

As a proof-of-concept, Gram-positive *S. aureus* served as model bacteria to prove the pH-dependent antibacterial property of the multilayer coatings. Primarily, the survival state and amounts of bacteria on the surfaces was observed by using CLSM (Fig. 5a). The bare Ti and PD layer suffered from serious bacteria invasion with a mass of dotted-green fluorescents on the surface, while the number of adherent bacteria was significantly decreased in the Ti-PD-DLSF and it showed better antibacterial ability at acidic condition, but some alive bacteria obviously attached on the surface, which tended to induced further infection (Fig. 5a). Between dip-coating and spin-coating CS barrier layer, the latter one displayed better bactericidal performances, and there were almost no bacteria on its surface at acidic condition. Presumably, at neutral condition (pH = 7.5), the anti-adhesion ability of Ti-PD-DLSF-SCS was attributed to intrinsic antibacterial property of CS chains. After the pH of medium decreased to 5.5, it showed improved bactericidal ability and almost all bacteria were erased, for the reason that collapse of CS barrier layer accelerated the release of bactericides. Among all groups, Ti-PD-DLSF-SCS exhibited the highest antibacterial efficiency due to the potential synergetic effects among CS, Gen and AgNPs^37,38^.

To further assess whether the local acidification served as a trigger to activate the multilayer coatings to be bactericidal, a WST cell viability test was utilized to quantify the cell amounts of adhesive and planktonic bacteria at different pH conditions (Fig. 5b,c). *S. aureus* could effortlessly invaded bare Ti surface at pH 5.5 and 7.5 with no apparent differences (Fig. 5b). Intriguingly, PD layer tended to approve bacteria colonized in acidic condition. As displayed in Fig. 5b, both Ti-PD-DLSF-DCS and Ti-PD-DLSF-SCS showed higher antibacterial capabilities compared to Ti-PD-DLSF group. Along with local acidification, the bacteria killing rate of Ti-PD-DLSF-DCS and Ti-PD-DLSF-SCS dramatically improved by 85.1% and 94.6%, respectively. The potential reason for this obviously pH-responsive antibacterial performance was that only CS layer contributed to bactericidal activity at neutral condition, while the released AgNPs showed synergetic antibacterial effect with CS at pH 5.5 due to the collapse of CS barrier layer. As displayed in Fig. 5c, Ti-PD-DLSF-SCS could kill the most planktonic bacteria (over 95%) at acidic condition, which further verified that the protonated amino groups of CS reinforced the repulsion between CS and AgNPs, and accelerated the release of AgNPs. Taken together, SCS-decorated multilayer coating showed excellent pH-dependent antibacterial ability. It was worth noting that DLSF layer exhibited relatively weak pH-dependent anti-adhesion and planktonic-killing property, derived from intrinsic structure of SF molecular, especially the content of β sheet structure. However, this DLSF layer still suffered from bacteria invasion and potentially caused cytotoxicity due to high amount of released Ag^+^. These results confirmed the vital role of pH-responsive CS barrier layer in constructing the smart antibacterial coatings.

### Long-Term Bacteria-Responsive Antibacterial Activity and Biofilm Assay

Having verified the multilayer coating with pH-dependent anti-adhesion and planktonic-killing behavior in the initial course of bacterial infection, we shifted our focus to examine the long-term bacterial-killing capability by SEM and Gram’s crystal violet staining assay. After initial adhesion, bacteria are apt to colonize the biomaterial’s surface, inducing local acidification around the infection sites, and severely develop into compact biofilm, which protects inner bacteria from attack of bactericides and releases free bacteria to infect other sites. So, the CS-decorated multilayer coating was expected to own long-term antibacterial and biofilm-inhibited capability, which played a vital role in its successful application.

The number and morphologies of adherent bacteria were first investigated by SEM observation (Fig. 6a). After co-culture with bacteria for 24 h, the bare Ti and PD layer suffered from severe bacterial invasion in both pH 5.5 and 7.5 growth medium and the attached bacteria maintained intact morphology and tended to be aggregated. Despite the existence of AgNPs/Gen complexes, some spherical and active cells were observed on Ti-PD-DLSF at neutral condition, whereas there were only a few bacteria with corrupted and distorted membranes on the PD-DLSF-DCS due to the membrane-disruption ability of CS chains, illustrating CS layer endowed a long-term bacteria-repellent capability to the multilayer coating. It should be noted that no intact bacteria attached on the surface of PD-DLSF-SCS, attributed to spin-coating CS layer contained plenty of active protonated amino groups, which acted as brushes to repluse bacteria. As expected, both Ti-PD-DLSF-DCS and Ti-PD-DLSF-SCS exhibited improved anti-adhesive capabilities at acidic condition. Very few cracked bacterial bodies stayed on Ti-PD-DLSF-DCS surface, while Ti-PD-DLSF-SCS presented a complete clean surface owing to initial burst release of bactericides. Visually, all the adherent bacteria on the Ti-PD-DLSF-DCS surface exhibited distorted and wrinkled membrane structure, suggesting the underlying synergetic effects of dissociative CS chains and Ag nanoparticles to destroy the bacterial membrane structure and eventually kill pathogens. Also, benefitting from amounts of protonated amino groups, spinning CS barrier layer displayed better dead bacteria-eliminated ability, maintaining the surface cleanliness. In addition, Fig. 6a clearly revealed the morphological and structural changes of CS barrier layer with the change of pH. In neutral solution, CS barrier layer maintained overall intact except some random distributed micropores. By contrast, the CS barrier layer underwent severe collapse, after exposing to acidic environment. Notably, at acidic condition, even the surface roughness highly improved, the SCS layer could still maintained integrity for 24 h co-culture with bacteria.

Considering the fact that a surface with lower amount of adhered biomass would have less tendency to form biofilm, Gram’s crystal violet staining assay was carried out to determine the total amount of biomass (including the bacteria and the components of biofilms) anchored on the multilayer coatings. As a positively-charged reagent, crystal violet can stain the living and dead cells as well as any negatively-charged film and matrix components. Thus, multilayer coatings that were not incubated in bacteria-containing growth media were also tested to eliminate the contribution of negatively-charged film components to the absorbance readings. Fig. 6c displayed biomass formation at the surface after one week incubation with bacteria. Quantitatively, the biofilm-associated crystal violet stains were collected by 95% ethanol solvent and spectrochemically measured. Higher OD value corresponded to stronger staining and thereby more accumulated biomass. Comparing to the bare Ti, the biomasses for Ti-PD-DLSF, Ti-PD-DLSF-DCS and Ti-PD-DLSF-SCS were remarkably reduced by 66.1%, 95.4% and 98.6%, respectively. Similar to the trends observed in the fluorescence staining and the SEM observation, DLSF layer exhibited relative biomass-inhibited capability and the amount of accumulated biomass on SCS barrier layer was the lowest, benefitting from pH-dependent and synergetic antibacterial properties. Figure 6c depicted optical pictures of the multilayer coatings with accumulated biomass. Obviously, both the bare Ti and PD layer were occupied by large areas of deep blue stains (mature biofilms), which coincided with quantitative results. Nevertheless, only a little blue spots could be identified on Ti-PD-DLSF-DCS surface, illustrating highly resistant to biofilm development, and Ti-PD-DLSF-SCS with a highly clean and smooth surface showed stirring biofilm-inhibited property.

### Biocompatibility Assay

The main goal of developing antimicrobic and tissue-integrating multilayer coating is to give the implants ability to kill bacteria, meanwhile, being able to approving the propagation and adhesion of tissue cells^39^. Thus, the cell viability assay was investigated to validate the toxicity of the pH-responsive multilayer coating. Initially, SEM was applied to observe the morphologies of attached cells (Fig. 7a), which was usually responsible for the following cellular functions and eventual differentiation. Then, cellular cytoskeleton morphologies in the following days were investigated *via* confocal observation (Fig. 7b). Generally speaking, cells would experience morphological changes to stabilize the cell−material interface after contacting biomaterials. As verified, anchored cells would undergo an intricate process to adhere and spread, consisting of initial attachment, filopodia development, cytoplasmic netting, interaction of cell mass and the progressing of peripheral cytoplasm^40^. After co-cultivating with MC3T3-E1 cells for 6 h, bare Ti acceptably approved cell adhesion and the cells were generally spindle shaped with pseudopodium (Fig. 7a), and less amounts of MC3T3-E1 cells with peripheral filopodia were attached on the surface of PD layer (Fig. 7c). However, coarsely circular cells without peripheral filopodia separately attached on the surface of Ti- PD-DLSF, presenting the worst spreading state (Fig. 7a,b). By sharp contrast, it could been noticed that cells on both Ti-PD-DLSF-DCS and Ti-PD-DLSF-SCS displayed better spreading state and flattened shape with a larger attached area, illustrating that the existence of CS barrier layer could efficiently block Ag release and highly improved cell attachment, spread and proliferation.

Furtherly, Fig. 7d showed the quantitative results of cellular proliferation by cck8 viability test, in which cellular proliferation occurred on all the samples. Compared with the bare Ti, thin PD layer supported osteoblast to adhere and proliferate, disclosing improved biocompatibility. Remarkably, the amount of attached cells on the Ti-PD-DLSF-SCS was ∼40% higher than that on the Ti-PD- DLSF at three time points. As verified in section 3.2, DLSF released approximate 1.8 times larger amount of Ag^+^ than that in DLSF-SCS in the initial 6 hour at neutral condition, so DLSF triggered relatively high cytotoxicity. In particular, the presence of CS, originated from the hard shell of insects and crustaceans with good biocompatibility, as a barrier layer remarkably improve the adhesion and proliferation of osteoblast^41^. Then, confocal observation was applied to examine the capacity of MC3T3-E1 cells to develop cytoskeleton on the multilayer coatings (Fig. 8a). After culturing for 1 d, relatively weak stress fibers were formed by F-actin on the surface of Ti-PD-DLSF, compared to the both CS barrier groups. The assembly of stress fibers continued in the following days, accompanied by replication, for all the observed groups. Particularly, bioactive SCS barrier layer led to networked cytoskeletons distributing throughout the cell bodies after 3 d cultivation, revealing that SCS barrier layer blocked the released of silver and stopped the cytoskeleton disturbance. Here, in agreement with previous study, low Ag^+^ concentration showed negligible effect on osteoblast adhesion, spreading and proliferation, and further improved the osteogenic differentiation in some extent.

To investigate the osteogenic differentiation potential of MC3T3-E1cells, *in vitro* ALP activities at 3-, 7- and 14-d were measured as an early hallmarker. ALP activity in osteoblasts is an important factor in promoting bone mineral formation and shows a scale of changes during osteoblastic differentiation. As illustrated in Fig. 8b, the normalized ALP activity for all groups increased from 3 d to 7 d. When cells were cultured on DCS layer for 7 d, they displayed high ALP values only next to Ti-PD-DLSF-SCS group, while the ALP activity expression on Ti-PD-DLSF was the lowest. Additionally, BCIP/NBT based ALP staining for the ALP activity on the entire surface was performed at 7 d (Fig. S3). The deeper the blue color, the stronger the expression of ALP. In accordance with quantitative results, the Ti-PD-DLSF-SCS displayed the best staining feature and thus the highest osteo-differentiation activity. The collagen secretion and mineral deposition, the intrinsic characteristics of osteogenic differentiation, were further estimated as osteogenic markers at the late stage. As depicted in Fig. 8c, Ti-PD-DLSF showed the lowest expression at both collagen secretion and mineral deposition, while Ti-PD-DLSF-SCS performed the strongest potential to induce osteoblast differentiation. Taken together, there three two factors benefitting the excellent osteogenetic activity of the CS-decorated multilayer coating: (1) spin-coating owned the CS barrier layer with higher surface smoothness and binding force, leading to a relatively hydrophilic and protein-adsorbed surface, which were benificial to cell spreading, proliferation and osteogenic differentiation; (2) As verified, AgNPs exerted cytotoxic effects on osteoblasts at high concentrations but induced cell activation (proliferation, cytokines release and chemotaxis) at low concentration by combining with DNA and activating the expression of osteogenic genes such as HIF-1α and IL-8. Indeed, current study also verified that AgNPs exhibited a restricted promotion effect on differentiation of osteoblast at concentrations lower than 10 μM^42^. As depicted in section 3.2, CS barrier layer efficiently slowed down the Ag^+^ release and created a low silver concentration medium for promoting MC3T3-E1 proliferation and differentiation; (3) CS was found to promote osteoblasts growth and mineral-rich matrix deposition, with positive influences on osteogenesis *in vitro* and *in vivo*. CS-related coatings were established to sustain human osteoblast with a spherical morphology, improve type I collagen expression, and also promote the differentiation of osteoprogenitor cells and bone formation^30,43^. So, the CS barrier layer in this work not only endowed pH-responsive property to the multilayer coating, but also promoted cellular attachment, proliferation and differentiation. Based on the above perspectives, we conjectured that the enhancement of cellular proliferation and osteogenesis in this well-designed multilayer coating stemmed from the synergetic effect of low concentration Ag and the bioactive CS barrier layer. Furthermore, it could be inferred that the comprehensive effect of CS and Ag was highly significant in achieving a balance between antibacterial ability and osteoinductive property, and constructing pH-dependent intelligent multilayer coating for intractable orthopedic infections.

## Experimental Section

### Coating design

The removal of sericin and *B. mori* cocoon silk dissolving process were implemented as the established procedures^44^. AgNO_3_ (4–240 mM) and Gen powders (0.4–2.4 g) were added into 2 mL of 5 wt % SF solution to produce a transparent SF/AgNO_3_/Gen mixture solution. The final AgNO_3_ and Gen concentration in the solution was 2–120 mM/L and 0.2–1.2 mg/L, respectively. Afterwards, an UV lamp (40 W, from Philips) was used to expose the SF/AgNO_3_/Gen solution under the ultra violet and the solution was cultured at room temperature for 1–5 h to form SF/AgNO_3_/Gen solution. 1 mg/mL CS solution preparation was conducted by dissolving chitosan and 1.0% (v/v) acetic acid in 0.15 M NaCl solution with pH adjustment by 1 M NaOH to 6.5 under magnetic stirring state.

**Figure 1:**
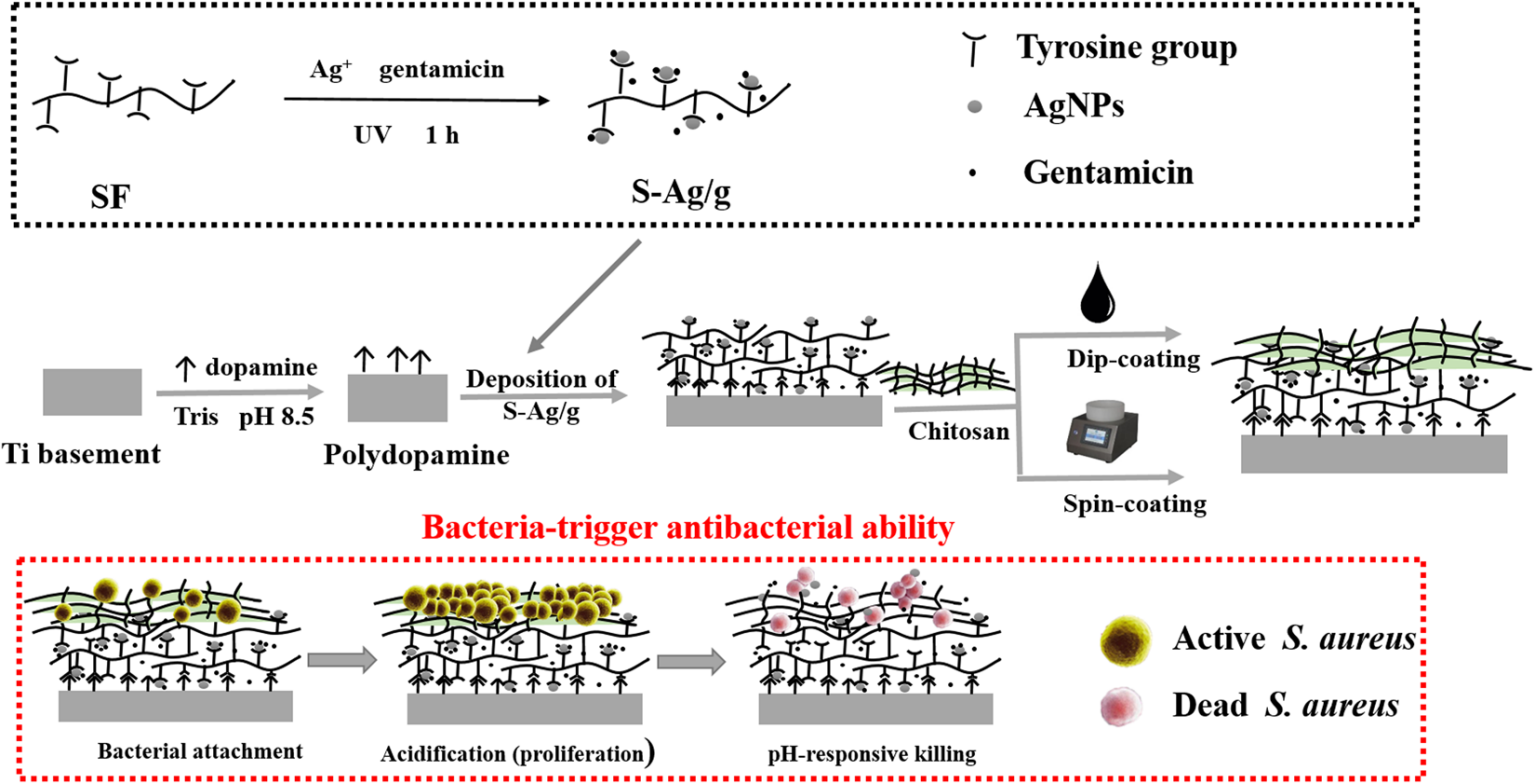
Conceptual illustration of constructing multifunctional multilayers through facile depositing processes. The depicted bacteria-responsive drug release mechanism is that attached bacterial proliferate and secrete lactic and acetic acid, which cause a local decrease in pH near the functional coating. The reduced local pH triggers the outmost CS barrier layer to be swelled and corrupted, and increase the release of AgNPs/Gen complexes loaded in inner SF layer to inhibit bacterial attachment and kill planktonic bacteria.

Bare Ti discs (10 mm × 10 mm × 0.5 mm) were mechanically polished up to 2000 grit and rinsed with ultrasonication in acetone, ethanol, and deionized water (DI) in sequence. Then, the discs were dry by blowing with purified nitrogen gas to get spotless and dehydrated surfaces for next decoration by polydopamine. Briefly, samples were placed into 2 mg/mL dopamine hydrochloride (Alfa Aesar) with Tris-HCl buffer (10 mM, pH = 8.5; Sigma) for 24 h under constantly vibrating in darkness at 37 °C, and then isolate the excess monomer and particles by thoroughly ultrasonication to produce polydopamine-decorated Ti (Ti-PD). PD-DLSF and PD-DLSF-CS functional coatings were obtained by a simple dipping-drying circular process in SF and CS solutions, respectively (Fig. 1a).

**Figure 2:**
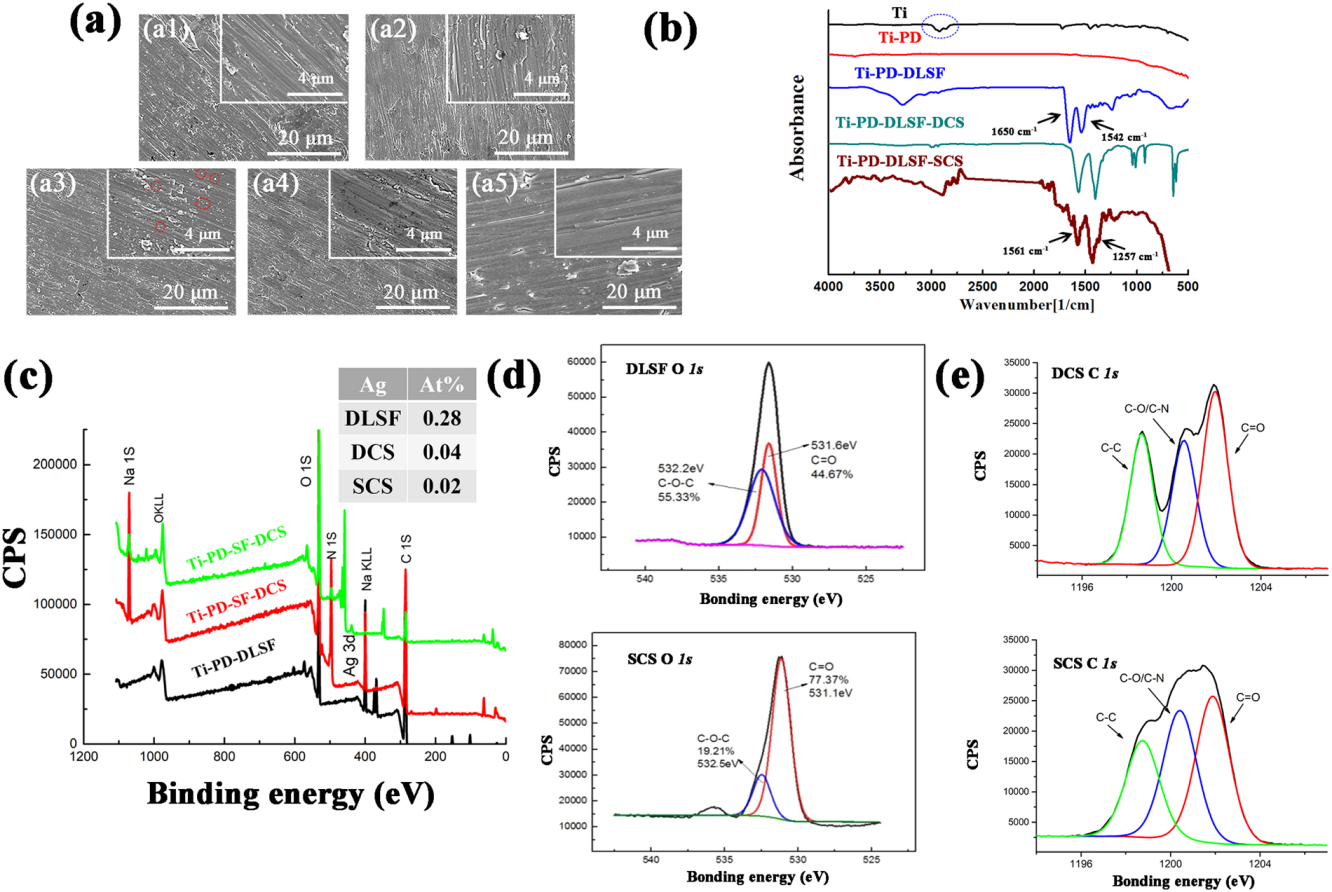
Physicochemical properties of the CS-decorated coating: (**a**) SEM images of Ti (a1), Ti-PD (a2), Ti-PD-DLSF (a3), Ti-PD-DLSF-DCS (a4), Ti-PD-DLSF-SCS (a5), the corresponding magnified images are shown as insets; (**b**) Micro-FTIR spectra; (**c**) XRD patterns; Corresponding core-level spectra for O 1s (**d**) and C 1s (**e**).

**Figure 3:**
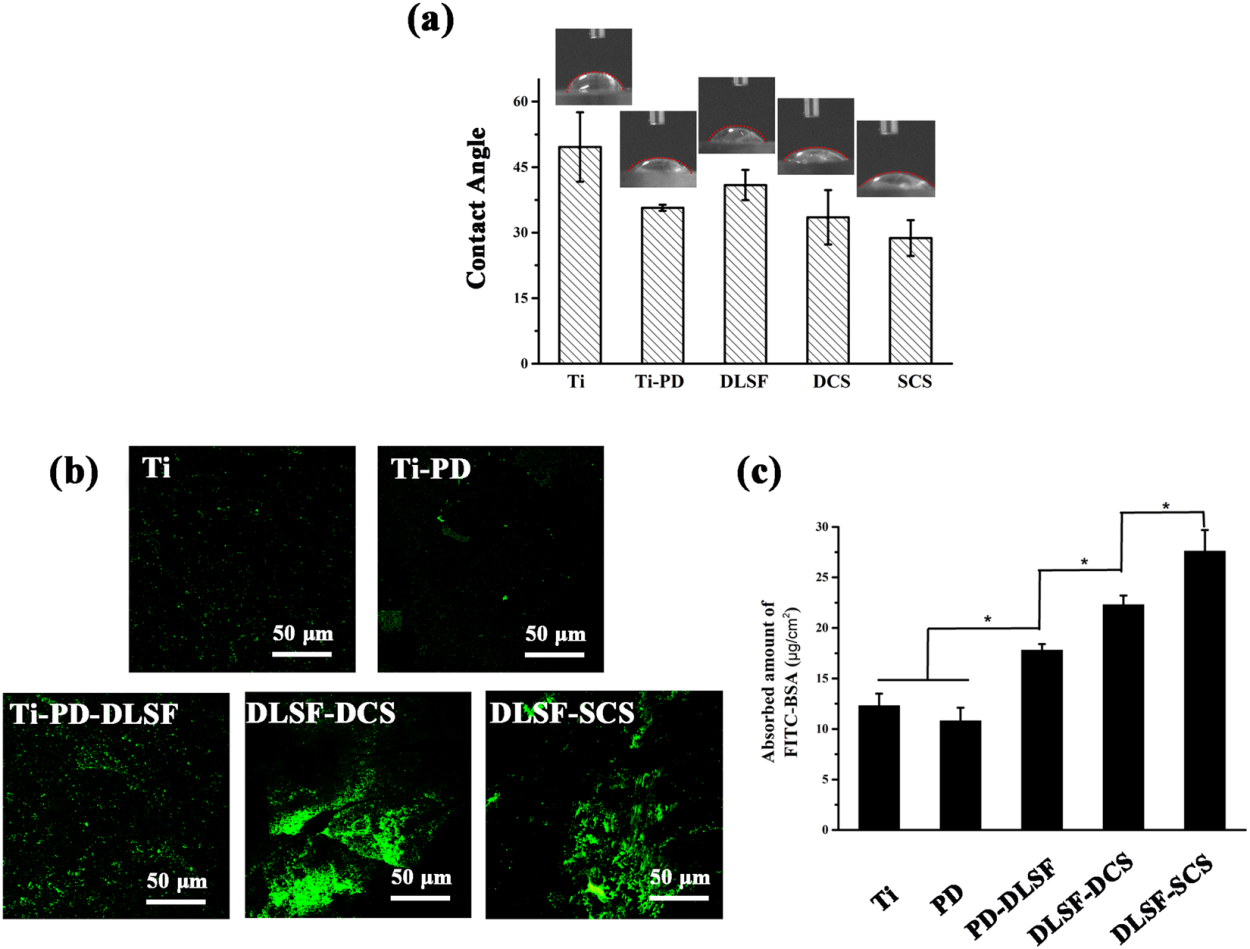
Surface properties of different samples: (**a**) Contact angle. (**b**) FITC-labeled BSA adsorption. (**c**) Quantitative calculation of adsorbed FITC-labeled BSA. *Represents p < 0.05.

**Figure 4:**
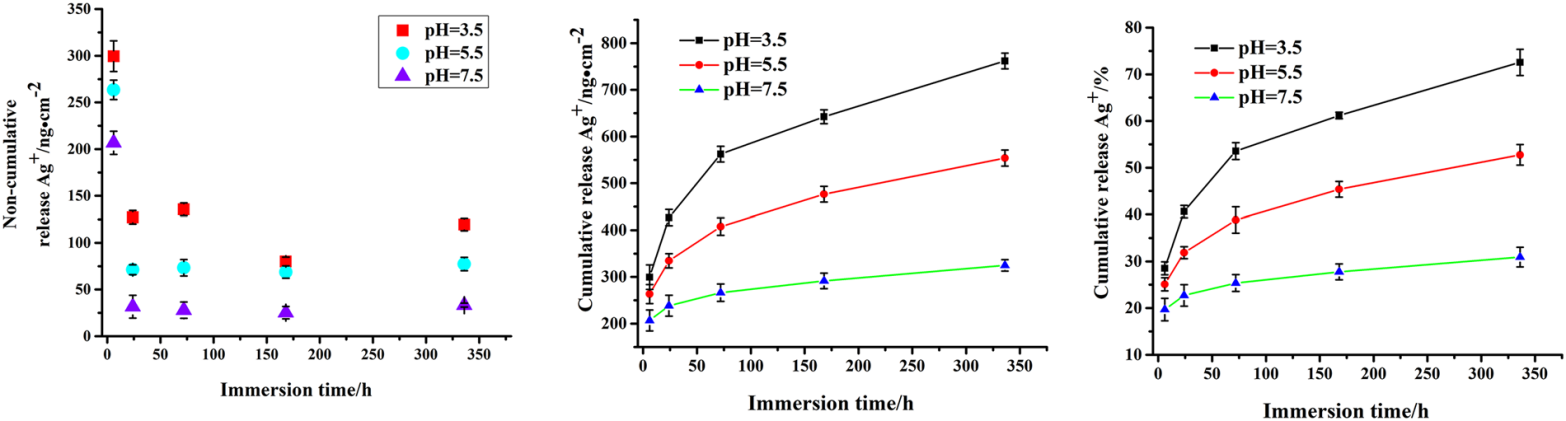
pH-dependent release behavior of silver in Ti-PD-DLSF-SCS group in PBS after immersion at 37 °C for 14 d.

**Figure 5:**
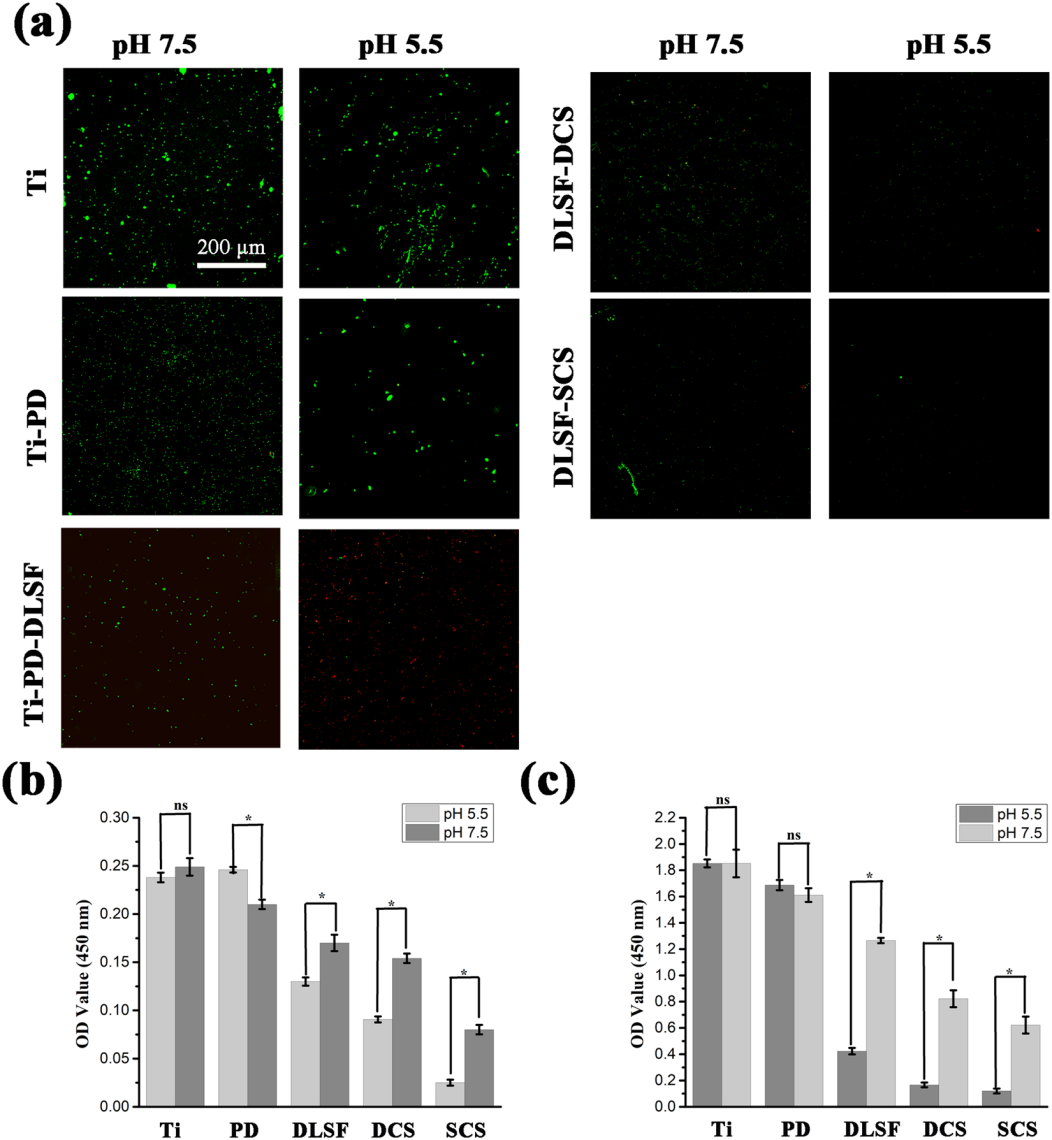
pH-dependent antibacterial property of CS-decorated coatings co-cultured with *S. aureus* suspension (10^8^ cells/mL) in different pH conditions (pH 7.5 and 5.5) for 4 h: (**a**) representative CLSM images of *S. aureus* on the surfaces; (**b**) comparison of the bacterial coverage and (**c**) killing efficiencies. *Represents p < 0.05.

**Figure 6:**
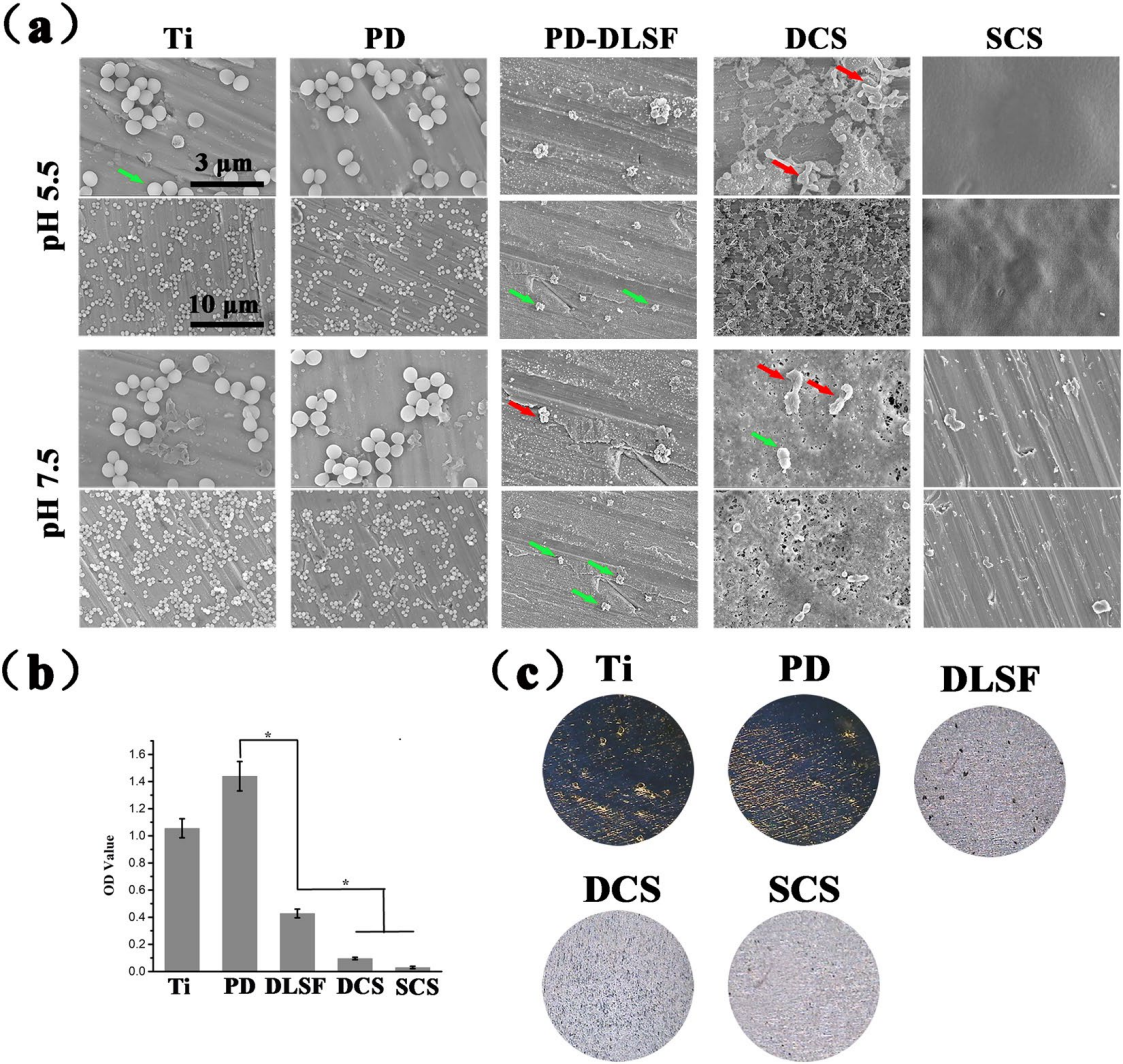
(**a**) Representative SEM images of *S. aureus* attached samples, which were incubated in growth medium containing 10^6^ mL^−1^ bacterial cells for 24 h. Green arrows indicate intact bacterial cells, red arrows indicate lesions and distortions on the cell membrane of microorganisms. Biofilm observation by Gram’s crystal violet staining: (**b**) biomass of adhered biofilms; (**c**) Visualization of biofilm formation.

**Figure 7:**
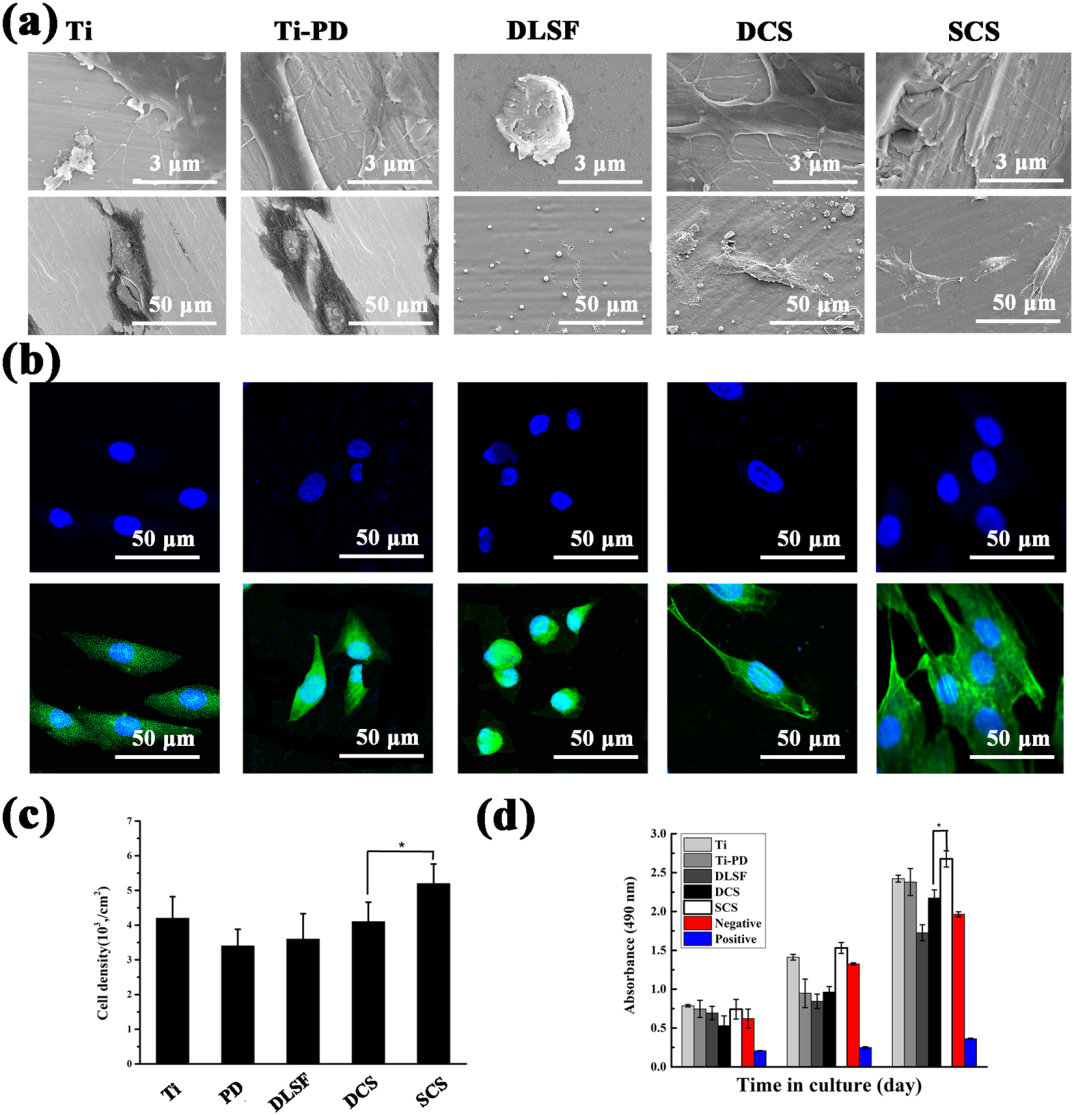
(**a**) SEM and (**b**) the staining of cytoskeletal actin fibers (green) and nuclei (blue) of adhering MC3T3s. (**c**) Quantitative evaluation of adhering cells on different sample surfaces at 6 h after seeding. (**d**) Proliferation of cells on different sample surfaces for 1, 3, and 7 days. *Represents p < 0.05.

**Figure 8:**
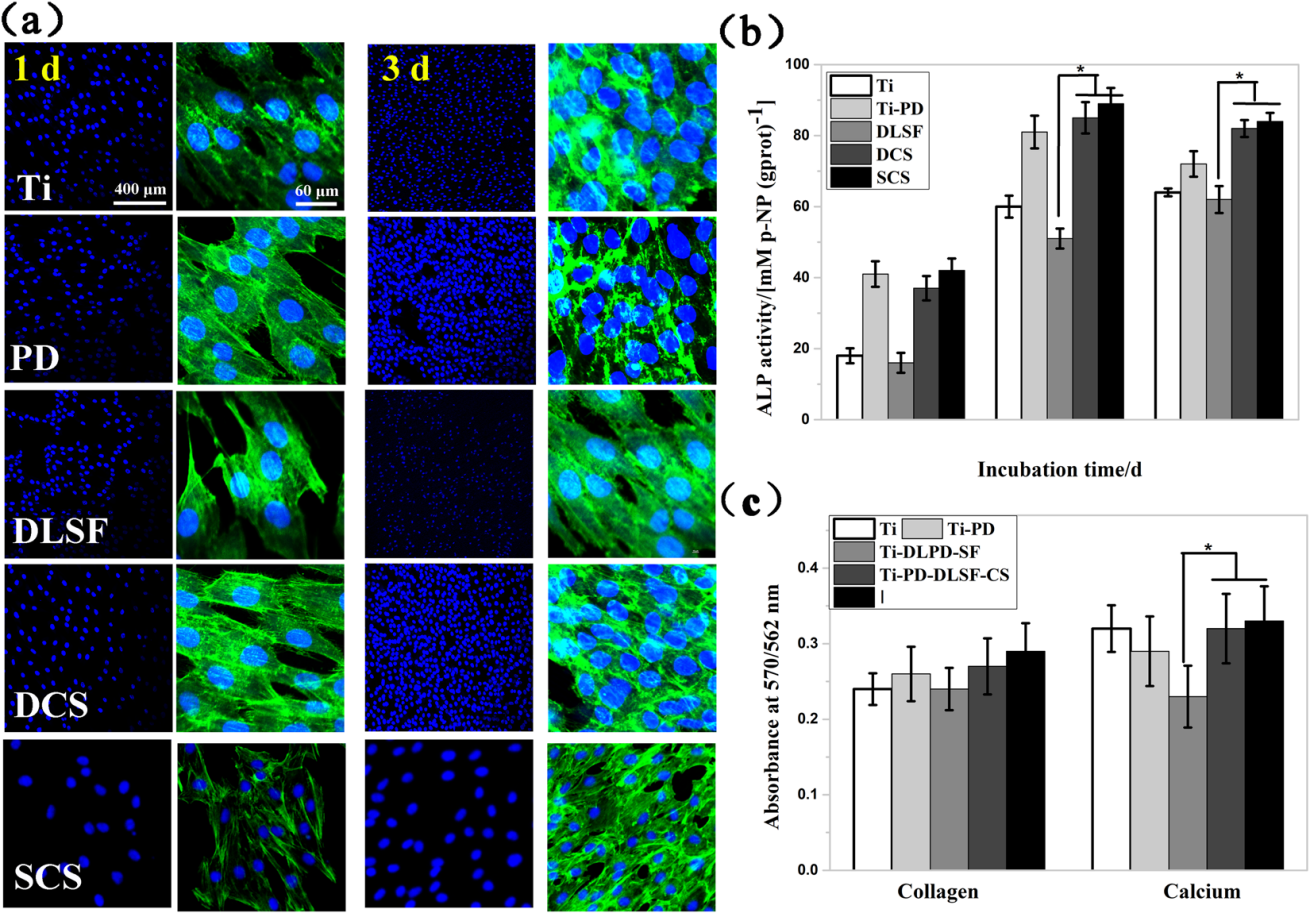
The cytocompatibility and osteoblastic functions of MC3T3-E1: (**a**) the staining of cytoskeletal actin fibers (green) and nuclei (blue); (**e**) the degree of ALP activity; (**f**) the quantification of collagen secretion and calcium deposition.

### Surface Characterization

Surface morphology was obtained by field-emission scanning electron microscopy (FE-SEM, S4800, Hitachi). X-ray photoelectron spectroscopy (XPS) was performed on an AXIS Ultra spectrometer (Kratos Analytical, Manchester, U.K.) with Al Kα excitation radiation (1486.6 eV). Fourier transform infrared (FTIR, Nicolet, Madison, WI) spectra were collected in transflective mode in the range of 700−4000 cm^−1^. The water contact angle on different specimens were determined by the sessile-drop water method, using an SL200B Contact Angle System (KINO, Norcross, GA). Protein adsorption test was accomplishment by immersing the functional coatings in FITC-labeled bovine serum albumin (FITC-BSA, 100 μg/mL, Sigma) for 60 min at 37 °C^45^.

### Ag^+^ Release Measurement

Samples (n = 3) were submersed in phosphate buffer solution (PBS, pH = 7.4) at 37 °C under static conditions. At the predetermined times, the leaching medium was collected and freshly added. Analysis was performed by using inductively coupled plasma mass spectrometry (ICP-MS; Agilent 7700×, U.S.). Besides, the total Ag content per sample was determined similarly after dissolving the particles in HNO_3_ (n = 3).

### Antimicrobial Activity Assays

A handy WST8-based microbial viability assay (Dojindo, Kumamoto, Japan) was used to investigate the ability of multilayer coatings in inhibiting invasion of bacteria by the established process. In a word, the production of microbial metabolism could react with WST-8 and an electron mediator, resulting in color change of LB solution, which could be measured by colorimetrical method.

After fixation with 2.5% (v/v) glutaraldehyde (GA) and dehydration in serial ethanol (50–100%), the morphologies of anchored bacteria was investigated by SEM observations.

Before SEM observing, the dried samples needed to be sputtered with gold to increase electrical conductivity. Additionally, *S. aureus* stayed on multi-layers coatings was stained by Live/Dead BacLight Bacterial Viability Kits (Invitrogen) to visualize the living state of bacteria.

Briefly, the coatings were rinsed, and 1 mL of stain mixture (6 μM SYTO 9; 30 μM propidium iodide (PI)) was added and kept in the dark for 15 min. The final fluorescent images formed by CLSM observation in XYZ scanning mode. The results were verified by repeatedly observing at more than 5 random regions.

The anti-biofilm ability of multi-layer coatings was investigated by incubating with bacteria (1 × 10^8^ CFU/mL) for 10 d. The culture medium was refreshed every 3 d. Firstly, the specimens were rinsed by PBS for 3 times to remove loose biofilm fragments, and then stained by 1% (w/v) crystal violet for 15 min. The specimens were washed by DI water until the waste solution was colorless. Finally, 95% (v/v) ethanol was used to elute biofilm-linked dyes by shaking at 37 °C for 30 min, which was measured by the absorbance at 570 nm.

### Cytocompatibility and Osteogenic Activity Assays

Cell Counting Kits (CCK-8, Dojindo) was used to quantify cell proliferation, as detailed elsewhere^46^, based on the measurement of mitochondrial activity. Additionally, attachment and spread out behavior of cells were observed by CLSM images of the stained actin cytoskeleton. For the immunofluorescence study, the samples were rinsed three times with PBS and fixed in 4% PFA/PBS. Fixed cells were permeabilized and counterstained with FITC-phalloidin (1:200, 40 min; Sigma) and DAPI (1:1000, 5 min). The early adhesion and spreading of MC3T3-E1 on samples were evaluated using SEM. At 6 h after seeding, cells were fixed with glutaraldehyde (2.5%, Sigma) for 2 h at RT. Then, cells were dehydrated in gradient ethanol (30, 50, 70, 80, 90, 95, and 100%) for 10 min per step. Afterward, samples were sputtered gold and examined under SEM.

For alkaline phosphatase (ALP) activity, after 3-, 7- and 14-d, cells on specimens were rinsed with PBS for 3 times and lysed with 1% Triton X-100 for 1 h, and then the cell lysis was mixed with substrate p-nitro phenylphosphate (pNPP; Jiancheng Biotech, Nanjing, China), transforming into p-nitro-phenol (pNP) by a ALP enzymatic reaction. Absorbance at 520 nm was read after colorimetric reaction. The ALP activity was normalized against total cellular proteins, as determined by BCA reaction, and expressed in U per gram of protein. Qualitatively, after fixation of cells with 4% PFA, the specimens were stained with BCIP/NBT ALP Color Development Kit (Beyotime, China). Histochemical dyes Sirius Red (SR, 0.1%; Sigma) and Alizarin Red S (ARS, 2%; Sigma), which combine with ECM collagen and calcium salts, were occupied to follow a 14 d and 28 d cultures, respectively. For staining, cells were fixed in 4% PFA and rinsed with PBS, and 500 μL of SR or ARS dye solution was added and incubated for 18 h or 15 min, respectively; after thorough washing, specimens were dried. Quantitatively, dyes were extracted using 50% 0.2 M NaOH/methanol (for SR) and 10% cetylpyridinium chloride (for ARS), and the samples were then read on a microplate at 570 or 562 nm.

## Electronic supplementary material

Supporting Information
