# Author Correction: Construction of Self-defensive Antibacterial and Osteogenic AgNPs/Gentamicin Coatings with Chitosan as Nanovalves for Controlled release

**DOI:** 10.1038/s41598-023-30139-4

**Published:** 2023-02-22

**Authors:** Wenhao Zhou, Yangyang Li, Jianglong Yan, Pan Xiong, Qiyao Li, Yan Cheng, Yufeng Zheng

**Affiliations:** 1 Academy for Advanced Interdisciplinary Studies, Peking University, Beijing, 100871 China; 2 Department of Advanced Materials and Nanotechnology, College of Engineering, Peking University, Beijing, 100871 China; 3 Department of Biomedical Engineering Materials Research Institute, The Huck Institutes of the Life Sciences, The Pennsylvania State University, University Park, State College, PA 16802 USA

Correction to: *Scientific Reports* 10.1038/s41598-018-31843-2, published online 07 September 2018

The original version of this Article contained errors. In Figure 6a, the PD-DLSF panel incorrectly showed two images of one sample. In Figure 8A, there was duplication between the actin staining image for Ti/1d and actin staining image for DLSF/3d.

The correct Figure 6 and 8 and their accompanying legends appear below.

**Figure 6:**
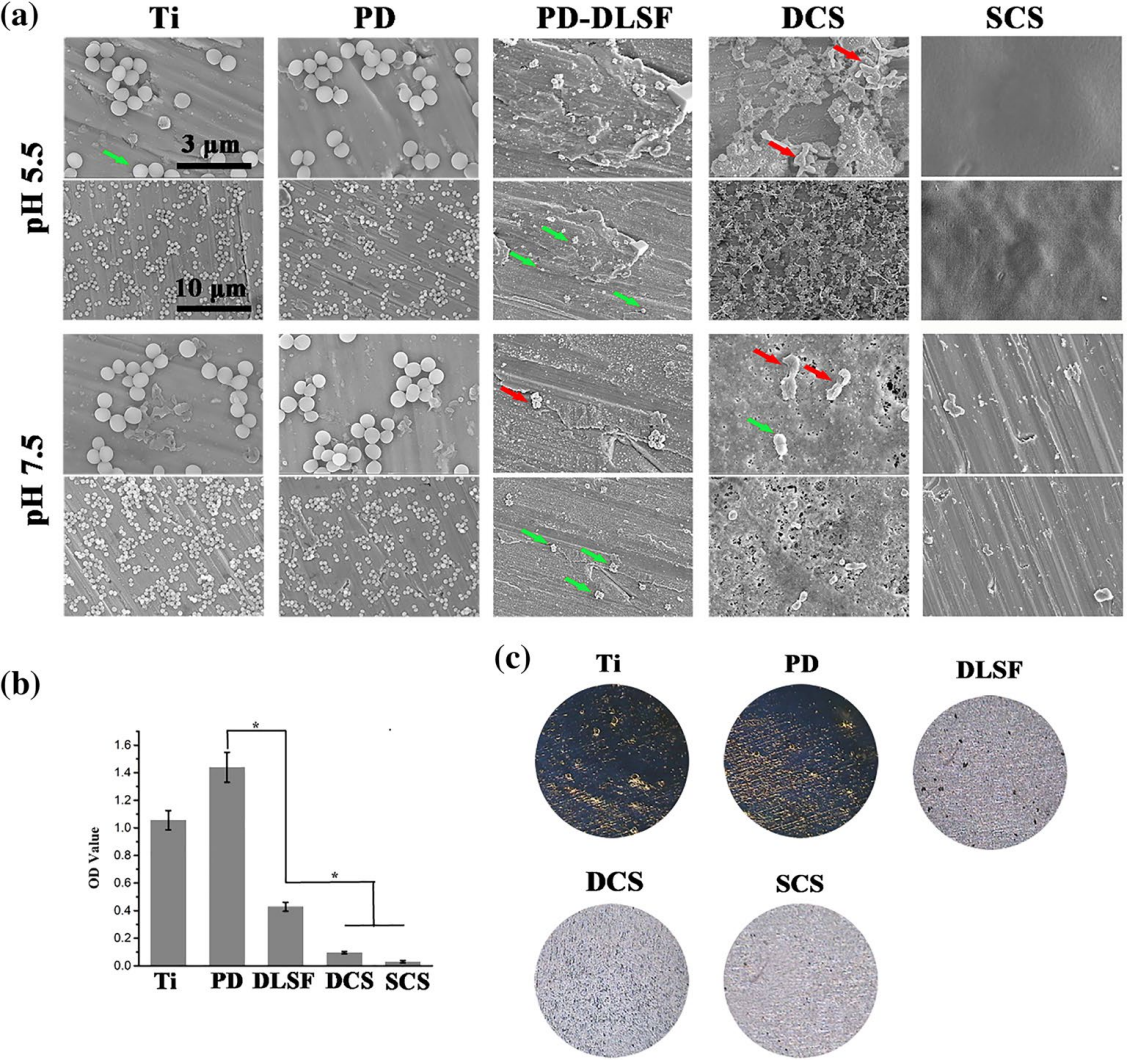
(**a**) Representative SEM images of *S. aureus* attached samples, which were incubated in growth medium containing 106 mL^−1^ bacterial cells for 24 h. Green arrows indicate intact bacterial cells, red arrows indicate lesions and distortions on the cell membrane of microorganisms. Biofilm observation by Gram’s crystal violet staining: (**b**) biomass of adhered biofilms; (**c**) Visualization of biofilm formation.

**Figure 8:**
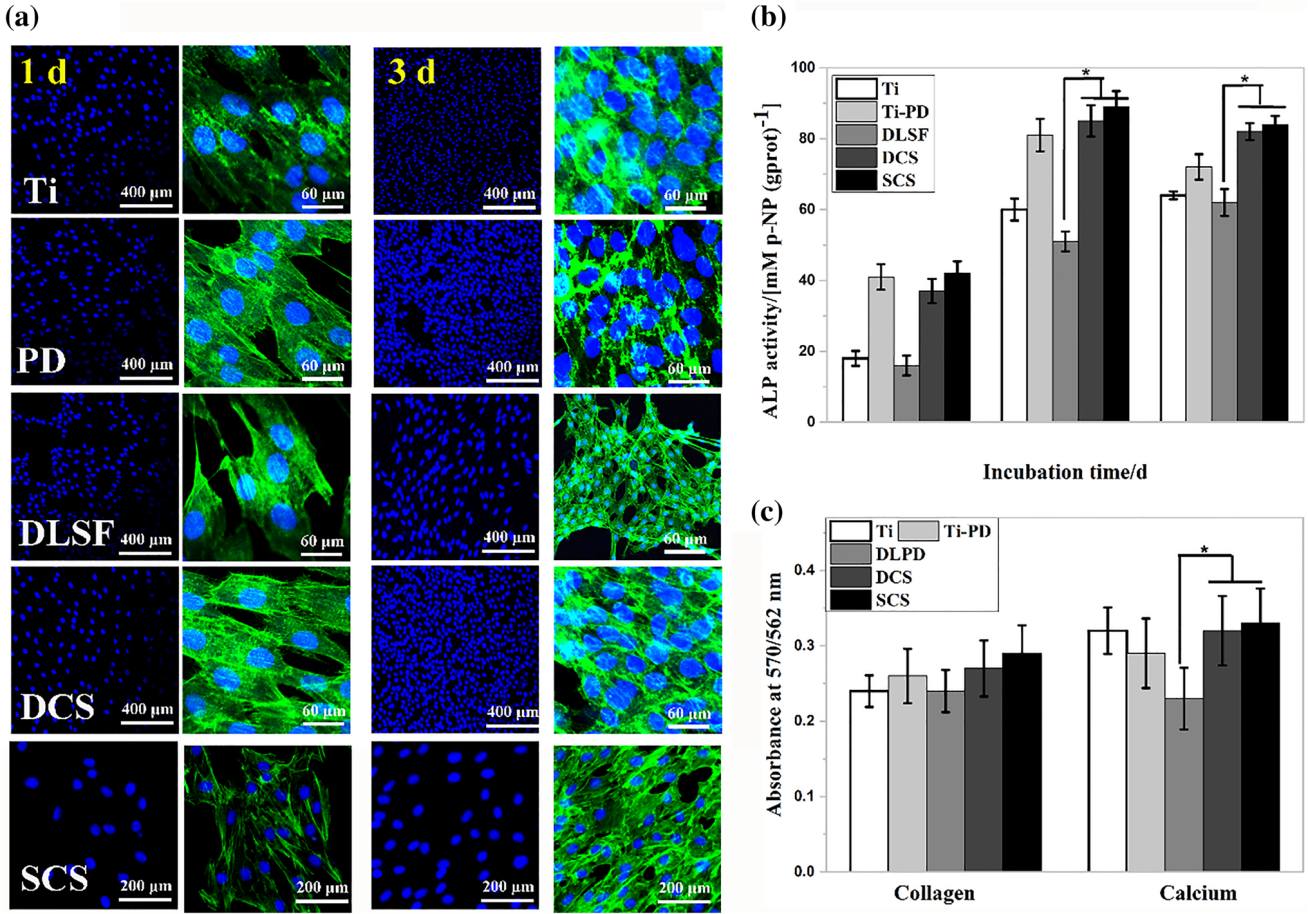
The cytocompatibility and osteoblastic functions of MC3T3-E1: (**a**) the staining of cytoskeletal actin fibers (green) and nuclei (blue); (**e**) the degree of ALP activity; (**f**) the quantification of collagen secretion and calcium deposition.

