# Supplementary material for: Construction of Self-defensive Antibacterial and Osteogenic AgNPs/Gentamicin Coatings with Chitosan as Nanovalves for Controlled release
Source: Sci Rep. 2018 Sep 7;8:13432. doi: 10.1038/s41598-018-31843-2 (PMC6128911; doi:10.1038/s41598-018-31843-2)
Supplement: Supplementary file 1 — Supporting Information [file 41598_2018_31843_MOESM1_ESM.docx]

# Construction of Self-defensive Antibacterial and Osteogenic AgNPs/Gentamicin Coatings with Chitosan as Nanovalves for Controlled release

Wenhao Zhou^a^, Yangyang Li^a^ , Jianglong Yan^a^, Pan Xiong^a^, Qiyao Li^c^, Yan Cheng^a,^*, Yufeng Zheng^a,b^

^a^*Academy for Advanced Interdisciplinary Studies, Peking University, Beijing 100871, China*

^b^*Department of Advanced Materials and Nanotechnology, College of Engineering, Peking University, Beijing 100871, China*

***Corresponding author**

Y. Cheng, Ph. D.

Academy for Advanced Interdisciplinary Studies,

Peking University, Beijing 100871, China

Tel&Fax: 0086-10-6275 3404

E-mail: [chengyan@pku.edu.cn](mailto:chengyan@pku.edu.cn)


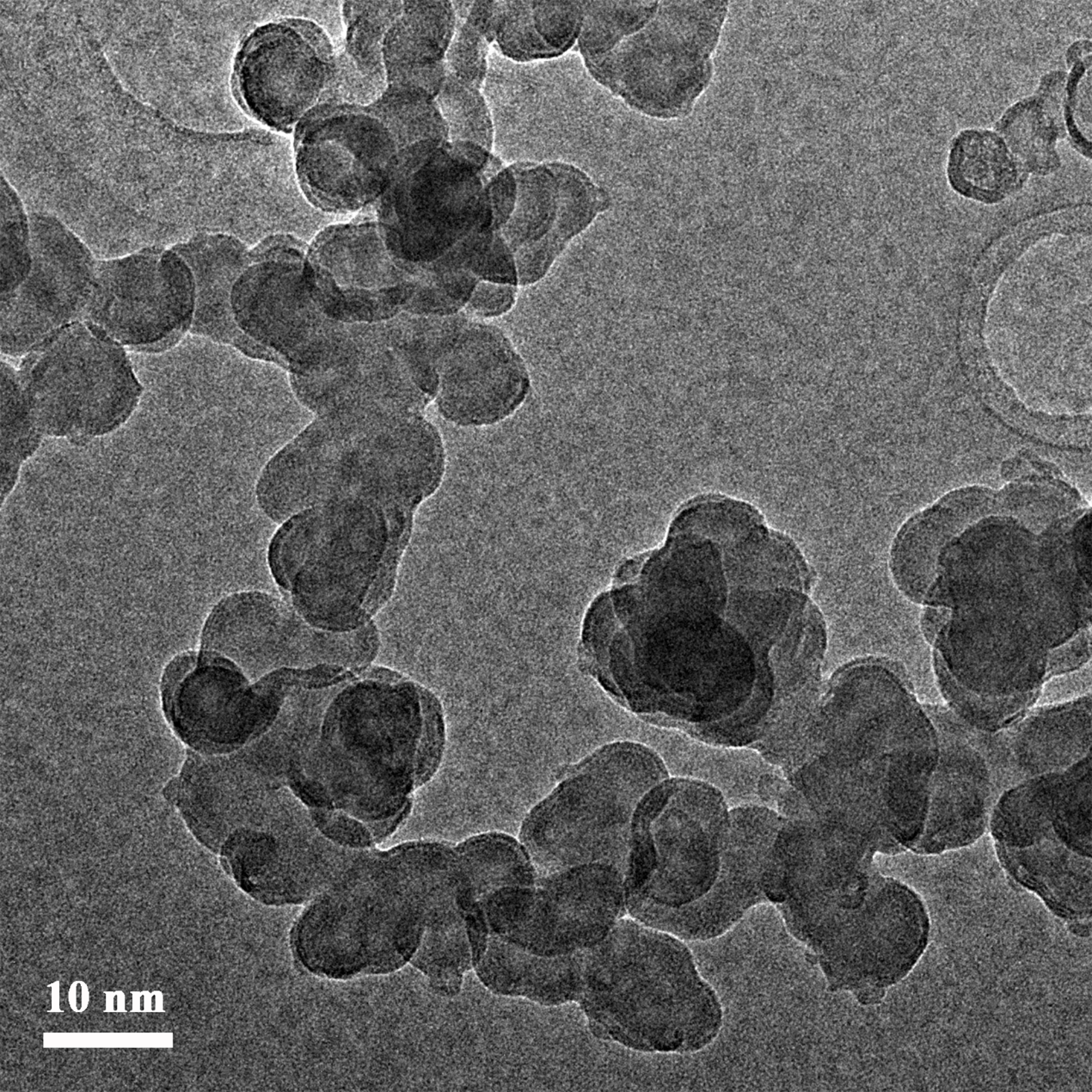


**Figure S1.** TEM observation of Ag nanoparticles.


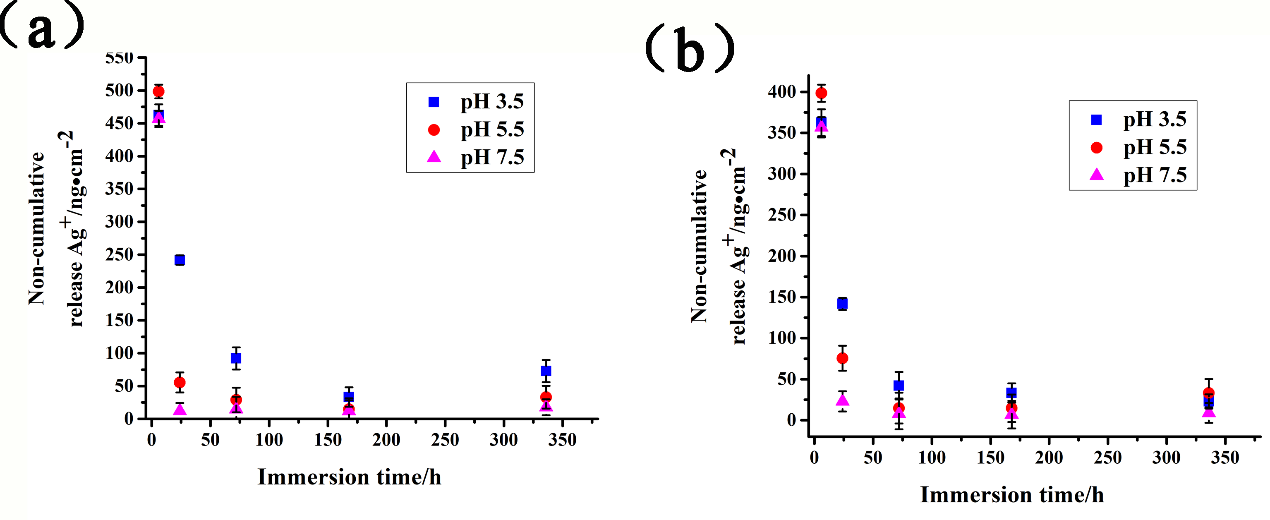


**Figure S2.** pH-dependent release behavior of silver in Ti-PD-DLSF (a) and Ti-PD-DLSF-DCS (b) after immersion at 37 °C for 14 d.


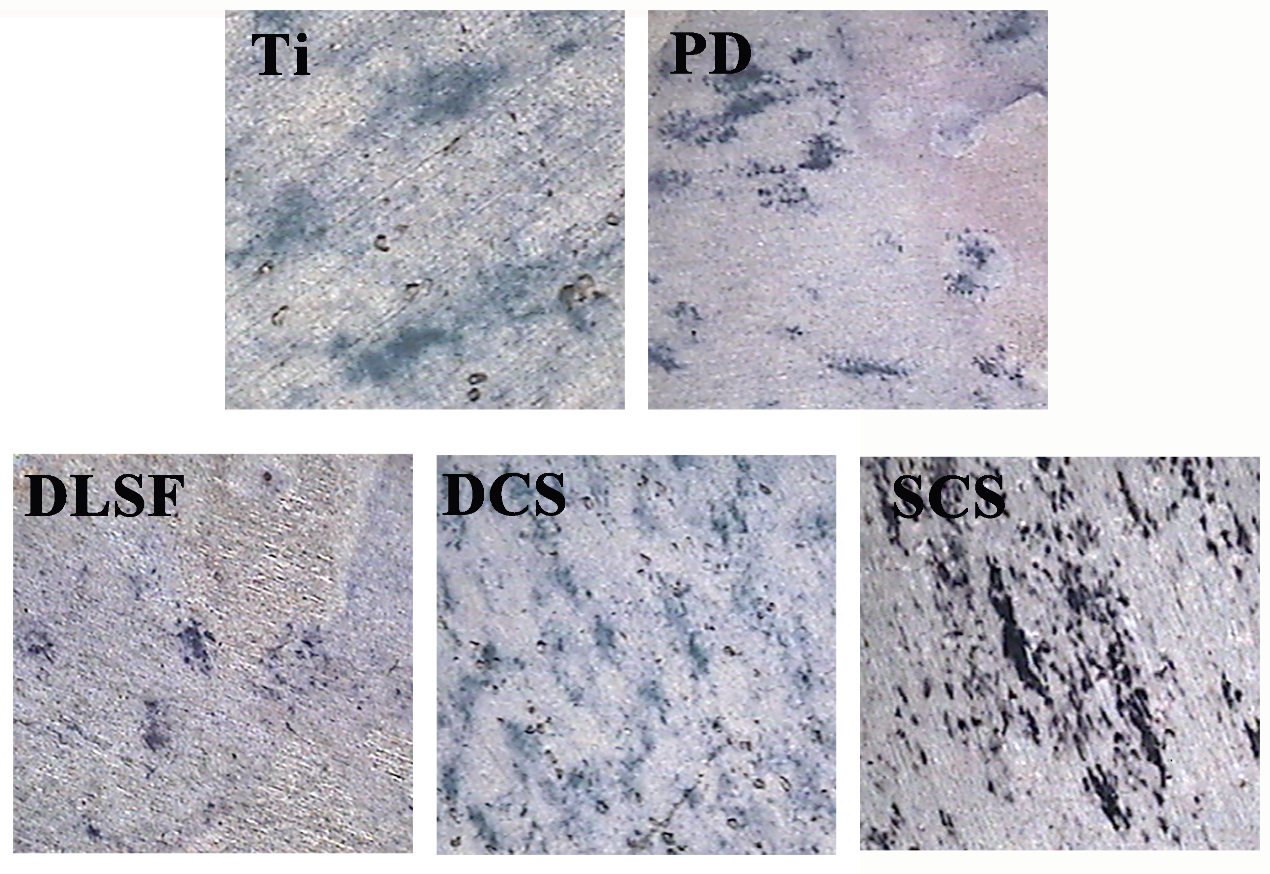


**Figure S3.** ALP staining of multi-layer coatings.
